# Early Warning and Prediction of Scarlet Fever in China Using the Baidu Search Index and Autoregressive Integrated Moving Average With Explanatory Variable (ARIMAX) Model: Time Series Analysis

**DOI:** 10.2196/49400

**Published:** 2023-10-30

**Authors:** Tingyan Luo, Jie Zhou, Jing Yang, Yulan Xie, Yiru Wei, Huanzhuo Mai, Dongjia Lu, Yuecong Yang, Ping Cui, Li Ye, Hao Liang, Jiegang Huang

**Affiliations:** 1 School of Public Health Guangxi Medical University Nanning China; 2 Guangxi Key Laboratory of AIDS Prevention and Treatment Guangxi Medical University Nanning China; 3 Life Science Institute Guangxi Medical University Nanning China; 4 Guangxi Colleges and Universities Key Laboratory of Prevention and Control of Highly Prevalent Disease Guangxi Medical University Nanning China

**Keywords:** scarlet fever, Baidu search index, autoregressive integrated moving average, ARIMA, warning, prediction

## Abstract

**Background:**

Internet-derived data and the autoregressive integrated moving average (ARIMA) and ARIMA with explanatory variable (ARIMAX) models are extensively used for infectious disease surveillance. However, the effectiveness of the Baidu search index (BSI) in predicting the incidence of scarlet fever remains uncertain.

**Objective:**

Our objective was to investigate whether a low-cost BSI monitoring system could potentially function as a valuable complement to traditional scarlet fever surveillance in China.

**Methods:**

ARIMA and ARIMAX models were developed to predict the incidence of scarlet fever in China using data from the National Health Commission of the People’s Republic of China between January 2011 and August 2022. The procedures included establishing a keyword database, keyword selection and filtering through Spearman rank correlation and cross-correlation analyses, construction of the scarlet fever comprehensive search index (CSI), modeling with the training sets, predicting with the testing sets, and comparing the prediction performances.

**Results:**

The average monthly incidence of scarlet fever was 4462.17 (SD 3011.75) cases, and annual incidence exhibited an upward trend until 2019. The keyword database contained 52 keywords, but only 6 highly relevant ones were selected for modeling. A high Spearman rank correlation was observed between the scarlet fever reported cases and the scarlet fever CSI (r_s_=0.881). We developed the ARIMA(4,0,0)(0,1,2)_(12)_ model, and the ARIMA(4,0,0)(0,1,2)_(12)_ + CSI (Lag=0) and ARIMAX(1,0,2)(2,0,0)_(12)_ models were combined with the BSI. The 3 models had a good fit and passed the residuals Ljung-Box test. The ARIMA(4,0,0)(0,1,2)_(12)_, ARIMA(4,0,0)(0,1,2)_(12)_ + CSI (Lag=0), and ARIMAX(1,0,2)(2,0,0)_(12)_ models demonstrated favorable predictive capabilities, with mean absolute errors of 1692.16 (95% CI 584.88-2799.44), 1067.89 (95% CI 402.02-1733.76), and 639.75 (95% CI 188.12-1091.38), respectively; root mean squared errors of 2036.92 (95% CI 929.64-3144.20), 1224.92 (95% CI 559.04-1890.79), and 830.80 (95% CI 379.17-1282.43), respectively; and mean absolute percentage errors of 4.33% (95% CI 0.54%-8.13%), 3.36% (95% CI –0.24% to 6.96%), and 2.16% (95% CI –0.69% to 5.00%), respectively. The ARIMAX models outperformed the ARIMA models and had better prediction performances with smaller values.

**Conclusions:**

This study demonstrated that the BSI can be used for the early warning and prediction of scarlet fever, serving as a valuable supplement to traditional surveillance systems.

## Introduction

Scarlet fever is an acute respiratory contagious disease caused by group A *Streptococcus pyogenes* infection and is classified as a category B notifiable infectious disease in China [1]. It is a seasonal disease that typically occurs during winter and spring, and no vaccine is currently available for prevention. Carriers and patients with scarlet fever are the primary sources of infection, which occurs mainly through airborne droplets. The population is generally susceptible, with children and adolescents being particularly vulnerable [2]. Clinical symptoms in infected patients are characterized by fever, pharyngitis, a diffuse bright red rash over the body, and skin flaking after the rash has disappeared [3,4]. The global incidence of scarlet fever has increased significantly since 2011, particularly in China, where a study has shown that the average annual incidence of scarlet fever between 2011 and 2016 was twice as high as that between 2004 and 2011 [5]. The resurgence of scarlet fever has emerged as a significant global public health issue [6]. Therefore, in order to comprehend the pattern and trend of scarlet fever outbreaks and facilitate the rational allocation of public health resources in China, a reliable prediction method is needed to identify the recent mode of the scarlet fever epidemic.

Currently, the autoregressive integrated moving average (ARIMA) model is one of the most commonly used time series methods and is extensively used in the early warning of infectious diseases [3,7-9]. The occurrence of scarlet fever exhibits seasonality and temporal correlation. The ARIMA model is capable of capturing such cyclic patterns and accounting for autocorrelation in time series data, thereby enhancing predictive precision. The emergence of disease is typically orchestrated by a confluence of diverse factors. By incorporating a multitude of exogenous variables, the ARIMA with explanatory variables (ARIMAX) model has shown enhanced capability in forecasting and delineating the progression of disease incidence [10]. The applicability of the ARIMA model in scarlet fever forecasting and early warning has been substantiated in China [3].

An increasing number of individuals are inclined to search for health-related information on the internet before seeking medical services, opening up the potential for early disease surveillance by monitoring fluctuations in the frequency of specific search keywords [11]. Internet-derived data revealed substantial potential in the application of infectious disease surveillance worldwide [12,13]. Google’s influenza monitoring data became available 2 weeks before official announcements in the United States [14]. In China, with over 904 million internet users, Baidu has the highest search engine market penetration and is used by over 90% of internet users, making it the most representative tool for measuring user behavior domestically [15]. Furthermore, the Baidu search index (BSI) can visually depict the changing trends in keyword search popularity. Studies have indicated that the BSI can be used for the early warning and prediction of communicable diseases, such as dengue fever [11], COVID-19 [15], and syphilis [16], in China.

However, scarlet fever is relatively underreported compared to influenza, and it is still unknown whether the BSI can enhance the predictive and early warning capacity for scarlet fever. Therefore, we developed an ARIMA model with scarlet fever cases and multiple ARIMAX models incorporating the BSI to investigate whether a low-cost, internet-based monitoring system can effectively complement traditional scarlet fever surveillance in China and provide a scientific basis for formulating strategies and policies for the prevention and control of infectious diseases.

## Methods

### Ethical Considerations

Data were obtained from the public National Health Commission of the People’s Republic of China database [17] and the researchers did not have access to individual patient details. The medical research ethics committee of the Guangxi Medical University determined that this research did not require ethics approval according to the following Chinese law: Notice by the National Health Commission, the Ministry of Education, the Ministry of Science and Technology, and the National Administration of Traditional Chinese Medicine of Issuing the Measures for Ethical Review of Life Science and Medical Research Involving Human Being (No. 4 of the National Health Commission, 2023).

### Data Sources

#### Epidemiological Data for Scarlet Fever

This study used monthly reported data from the National Health Commission of the People’s Republic of China [17] covering 140 months from January 2011 to August 2022. The data is publicly available for research purposes, no personal information is involved, and the collection of monthly reported data has been fully completed.

#### BSI Data

We obtained real-time search data and keywords for the same period from the BSI [18]. The keyword search index was based on the volume of information search queries conducted by Baidu search users and achieved through a corresponding calculation process. We collected data for relevant keywords spanning from January 2011 to August 2022 and converted them into monthly counts for subsequent analysis using Microsoft Excel 2019 (Microsoft Corporation).

### Keyword Selection and Analysis

#### Establishing a Keyword Database

The effectiveness of the model in predicting outbreaks using internet-derived data relies on selecting appropriate keywords for model fitting. Thus, selecting relevant keywords is crucial for ensuring accurate model fitting. Keywords related to scarlet fever with complete time series were collected using the following three approaches: (1) obtaining scarlet fever–related syndrome words using the ChinaZ website [19], (2) obtaining words related to scarlet fever according to the Baidu index demand map [20], and (3) mining keywords using semantic analysis of scarlet fever through the Baidu encyclopedia [21] and the Baidu health medical dictionary [22]. Eventually, the keywords were divided into 4 categories in Microsoft Excel 2019, namely, scarlet fever comprehensive category, etiology, symptoms, and prevention and treatment [23-25].

#### Keyword Exclusion Criteria

Keywords were excluded based on the following criteria: (1) keywords irrelevant to the epidemic or clinical information of scarlet fever, (2) keywords with a Spearman rank correlation (r_s_) <0.6 between scarlet fever reported cases and the BSI or *P>*.05 [26], and (3) keywords with a maximum cross-correlation coefficient <0.5 [27]. The analyses were performed using SPSS 26.0 (IBM).

### Construction of the Comprehensive Search Index

After the correlation analysis, a scarlet fever comprehensive search index (CSI) [26,27] was calculated as follows:



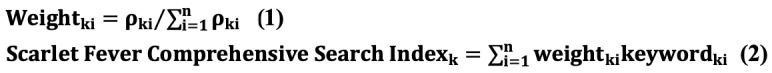



where k is potential time lag in months, n is the number of keywords contained in each time lag, _ki_ is the Spearman correlation coefficient for keyword i with a specific time lag k, and weight_ki_ and keyword_ki_ are the keyword’s weight and BSI in a lag period, respectively.

### Model Construction and Evaluation

#### Model Description

The ARIMA model is commonly used for forecasting the incidence of scarlet fever. Considering the time autocorrelation between the data points and the seasonal transmission pattern of scarlet fever, the model expression is ARIMA(p,d,q)(P,D,Q)_(S)_ [10], where d and D represent the nonseasonal and seasonal difference orders, respectively; p and q represent the autoregressive and moving average orders, respectively; P and Q represent the seasonal autoregressive and moving average orders, respectively; and S is the period of the sequence [8]. ARIMAX is a multivariable version of ARIMA that combined scarlet fever reported cases with the scarlet fever CSI, thus using the BSI as the external variable [26]. The construction and evaluation of the model were performed using R 4.2.0 (R Foundation for Statistical Computing), following the described process.

#### Stationarity Tests for Time Series

We plotted the time series of scarlet fever reported cases and the scarlet fever CSI to observe long-term trends from January 2011 to August 2022. The stationarity was tested using the augmented Dickey-Fuller (ADF) and Ljung-Box tests [10]. Nonstationary sequences were transformed into stationary sequences by difference and exponential transformation.

#### Splitting the Training and Testing Sets

Based on the stationary sequences, we divided the scarlet fever reported cases and the CSI data into the training sets for model construction running from January 2011 to August 2021 and the testing sets for model prediction running from September 2021 to August 2022. To evaluate the impact of the COVID-19 pandemic on scarlet fever, we conducted a subgroup analysis with the training sets running from January 2011 to December 2018 and the testing sets running from January 2019 to August 2022.

#### Model Selection

We used the scarlet fever reported cases training set to construct the ARIMA model, and both the scarlet fever reported cases and CSI training sets for the ARIMAX model. We used the *auto.arima* function for automatic selection and manual selection through examination of the autocorrelation function (ACF) and partial autocorrelation function (PACF) plots. First, the ARIMA and ARIMAX models were based on the *auto.arima* function and the minimum Akaike information criterion (AIC). Next, the orders and ranges of the ARIMA model were determined based on the ACF and PACF plots. We performed multiple fittings and searched for the optimal ARIMA(p,d,q)(P,D,Q)_(S)_ model using the minimum AIC [26]. Then, we plotted the cross-correlation function (CCF) plot between scarlet fever and the CSI to determine the appropriate lag order for the ARIMAX model [8]. If the coefficient for a specific lag order exceeded 2 SD, the CSI at that lag order was considered correlated with scarlet fever. Finally, all models were tested using the least squares method (LSM; *P*<.05 indicated model estimation was significant) and parameter estimation (*P*<.05 indicated a statistically significant parameter). The model diagnosis was performed using the residuals Ljung-Box test (*P*>.05 indicated the model variables were independent) [10,26]. After passing the tests, the models proceeded to the prediction section.

#### Evaluation of the Model Fit

The model fit was assessed using R^2^, AIC, root mean square error (RMSE), mean absolute error (MAE), and mean absolute percentage error (MAPE). A higher R^2^ value and lower values of the remaining variables indicated a better fit of the model.

#### Evaluation of the Model Prediction

**Figure 1:**
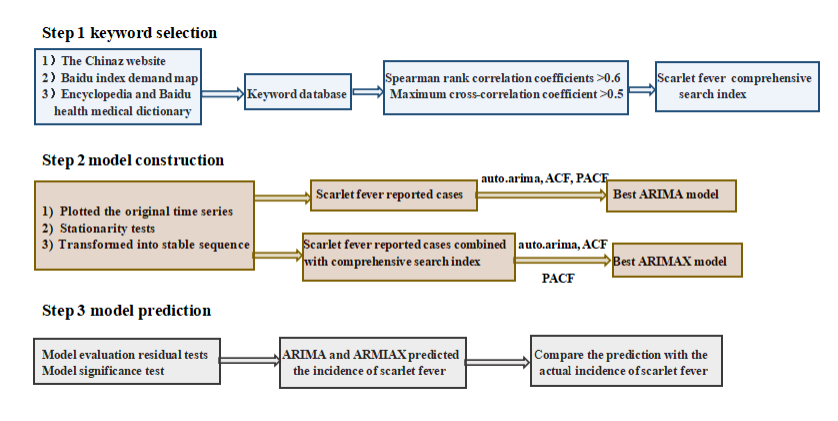
Research flowchart. ACF: autocorrelation function; ARIMA: autoregressive integrated moving average; ARIMAX: autoregressive integrated moving average with explanatory variable; PACF: partial autocorrelation function.

The ARIMA and ARIMAX models were used for predicting the incidence of scarlet fever with the testing sets, and the predictions were compared to the actual incidence. We evaluated the model prediction effects through MAE, RMSE, and MAPE. Smaller values indicated better predictions. The flow chart of this study is shown in Figure 1. All tests were 2-sided, and *P<*.05 indicated significance.

## Results

### Descriptive Analysis

From January 2011 to August 2022, the average monthly incidence of scarlet fever was 4462.17 (SD 3011.75) cases, with an annual incidence of 53,546.06 cases. The annual incidence of scarlet fever showed an upward trend until 2019, reaching a peak of 83,028 cases in 2019. The keyword database comprised 52 keywords categorized into the following categories: scarlet fever comprehensive category, etiology, symptoms, and prevention and treatment (Multimedia Appendix 1).

### Correlation Analysis Between the Baidu Search Index and Scarlet Fever

**Figure 2:**
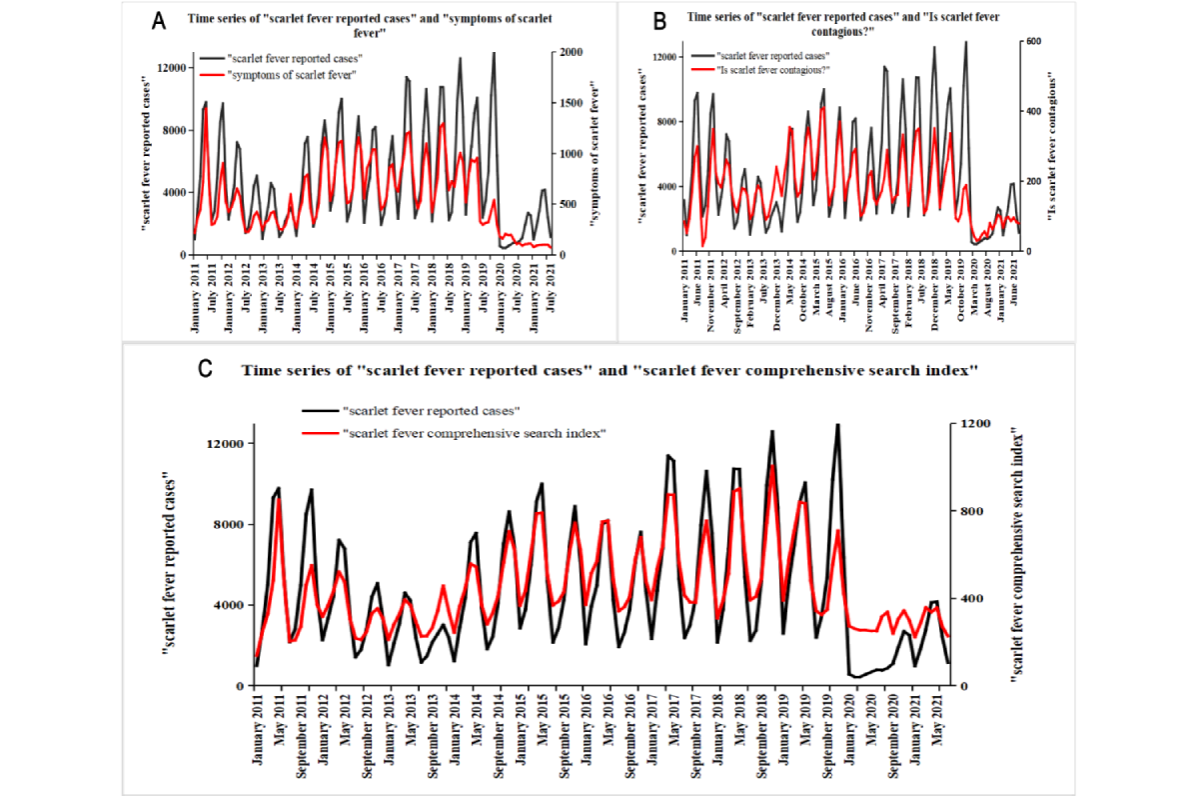
Time series between scarlet fever reported cases and (A) the keywords “symptoms of scarlet fever,” (B) the keywords “Is scarlet fever contagious?” or (C) the scarlet fever comprehensive search index.

The results of the Spearman rank correlation analysis revealed a strong temporal correlation between 6 keywords from the BSI and scarlet fever reported cases (r_s_>0.6; Table 1). The remaining keywords showed weak correlation or no statistical difference. Plotting the time series of high-correlation keywords, such as “猩红热症状 (symptoms of scarlet fever)” (Figure 2A) and “猩红热传染吗 (Is scarlet fever contagious?)” (Figure 2B), and comparing them with scarlet fever reported cases, the overall trends of the two keywords were consistent with the actual incidence.

**Table 1:** Spearman rank correlation analysis between the monthly Baidu search index keywords and scarlet fever reported cases.

Keywords^a^	r_s_^b^	*P* value	Search volume, mean (SD)
猩红热传染吗 (Is scarlet fever contagious?)	0.834	<.001	182.197 (89.909)
猩红热症状 (symptoms of scarlet fever)	0.818	<.001	565.129 (330.355)
猩红热图片 (scarlet fever pictures)	0.750	<.001	235.494 (111.399)
猩红热 (scarlet fever)	0.746	<.001	1482.185 (634.117)
猩红热症状图片 (scarlet fever symptoms pictures)	0.698	<.001	77.723 (49.489)
猩红热的症状 (scarlet fever symptoms)	0.649	<.001	92.044 (37.554)

^a^Keywords were presented in a Chinese (English) format.

^b^r_s_: Spearman rank correlation.

### Cross-Correlation Analysis and the CSI

Cross-correlation analysis was performed between keywords with r_s_>0.6 and scarlet fever reported cases. We selected 6 keywords with a maximum cross-correlation coefficient >0.5 (*P*<.001) within the lag range (Table 2). We calculated the weight of each keyword using a formula and used the weight to construct the scarlet fever CSI by aggregating the BSI of each keyword. Spearman rank correlation analysis showed a high correlation between scarlet fever reported cases and the scarlet fever CSI (r_s_=0.881).

**Table 2:** Cross-correlation analysis between scarlet fever reported cases and Baidu search index keywords.

Keywords^a^	Lag (months)	Maximum CCF^b^ (SE)
猩红热传染吗 (Is scarlet fever contagious?)	0	0.813 (0.088)
猩红热 (scarlet fever)	0	0.827 (0.088)
猩红热症状 (symptoms of scarlet fever)	0	0.809 (0.088)
猩红热图片 (scarlet fever pictures)	0	0.729 (0.088)
猩红热症状图片 (scarlet fever symptoms pictures)	0	0.678 (0.088)
猩红热的症状 (scarlet fever symptoms)	0	0.614 (0.088)

^a^Keywords were presented in a Chinese (English) format.

^b^CCF: cross-correlation function.

### Model Construction and Prediction Evaluation

#### Stationarity Tests for Time Series

Plotting the time series of scarlet fever reported cases and the scarlet fever CSI from January 2011 to August 2022 showed irregular fluctuating patterns (Figure 2C). The stationarity was tested using the ADF and Ljung-Box tests. The results of the ADF test indicated that the time series of scarlet fever reported cases (*P*=.70) and the scarlet fever CSI (*P*=.90) were nonstationary; they were transformed into stationary series by first order differencing (Multimedia Appendix 2), after which they passed the ADF test (*P*=.01). The 2 series were applied to construct and predict the incidence of scarlet fever based on the Ljung-Box test (*P*<.001).

#### Model Selection

The models ARIMA(4,0,0)(0,1,1)_(12)_ and ARIMAX(1,0,2)(2,0,0)_(12)_ were constructed according to the *auto.arima* function and the AIC minimum principle.

**Figure 3:**
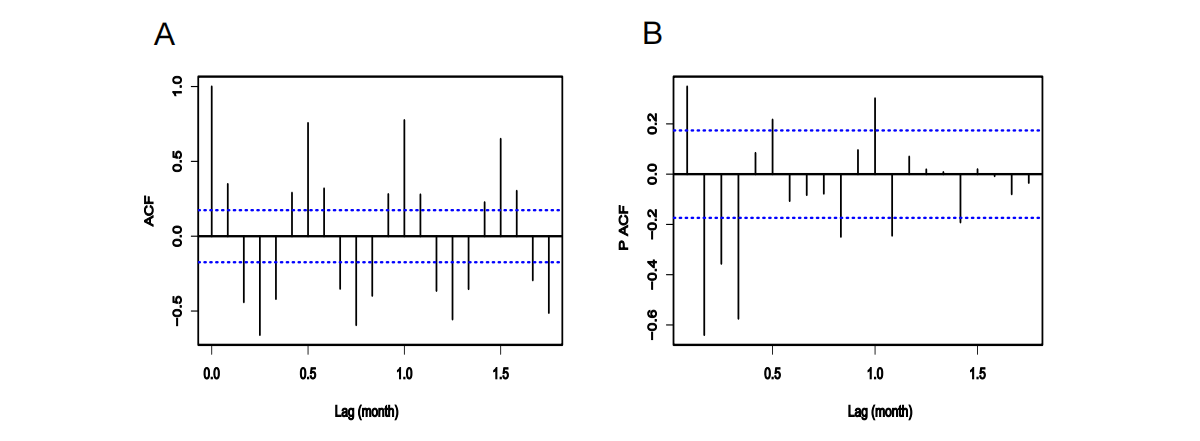
The (A) ACF and (B) PACF plots of scarlet fever. ACF: autocorrelation function; PACF: partial autocorrelation function.

The ACF and PACF plots of the stationary scarlet fever series showed that the ACF trailed off and the PACF was truncated at a lag of 4 months (Figure 3). The ACF plot exhibited a seasonal pattern with a period of 12 months, and the sequence was different by first order and passed the ADF (*P*=.01). Therefore, the parameters of the ARIMA model were p=4, q=0, d=0, D=1, and S=12. The model was fitted using different values for the parameters ranging from 0 to 2 in a stepwise manner from lower to higher orders [10]. After conducting combination tests, the model ARIMA(4,0,0)(0,1,2)_(12)_ was selected based on the minimum AIC of 1947.03, and the residuals conformed to a white noise sequence (χ^2^=0.145; *P*=.70).

The CCF plot considered the mutual relationship within a lag of 12 months, revealing that the CSI at a lag of 0 months exhibited the strongest correlation with scarlet fever (Multimedia Appendix 3). The stationary time series of CSI at a lag of 0 months was used as the input sequence for constructing the ARIMA(4,0,0)(0,1,2)_(12)_ + CSI (Lag=0) and ARIMA(4,0,0)(0,1,1)_(12)_ + CSI (Lag=0) models. All the aforementioned models passed the LSM and the residuals Ljung-Box tests (Table 3). The results of the model parameter estimation are presented in Multimedia Appendix 4. Although not every parameter exhibited significance, the estimated SEs of the parameters were relatively small, indicating minimal variability. Based on the minimum AIC principle and comprehensive evaluation of the fitting metrics (Table 3), we finally selected the ARIMA(4,0,0)(0,1,2)_(12)_ (Model 1), ARIMA(4,0,0)(0,1,2)_(12)_ + CSI (Lag=0) (Model 2), and ARIMAX(1,0,2)(2,0,0)_(12)_ (Model 3) models for predictive analysis.

**Table 3:** Evaluation of the model fit and diagnosis.

Model		ADF^a^, *P* value	Diagnosis				Fit				
			LSM^b^, *P* value	Ljung-Box test		AIC^c^		R^2^	RMSE^d^	MAE^e^	MAPE^f^
				Chi-square	*P* value						
ARIMA(4,0,0)(0,1,2)_(12)_		.01	<.001	0.15	.70	1947.03		0.87	924.6	626.44	709.00
ARIMA(4,0,0)(0,1,2)_(12)_ + CSI (Lag=0)		.01	<.001	0.01	.94	1894.89		0.91	755.76	545.01	588.56
ARIMAX(1,0,2)(2,0,0)_(12)_		.01	<.001	0.03	.86	2083.73		0.90	815.90	635.43	508.98
ARIMA(4,0,0)(0,1,1)_(12)_		.01	<.001	0.16	.69	1950.43		0.85	1006.71	694.06	876.77
ARIMA(4,0,0)(0,1,1)_(12)_ + CSI (Lag=0)		.01	<.001	0.01	.92	1893.99		0.91	779.49	563.22	643.87

^a^ADF: augmented Dickey-Fuller.

^b^LSM: least squares method.

^c^AIC: Akaike information criterion.

^d^RMSE: root mean square error.

^e^MAE: mean absolute error.

^f^MAPE: mean absolute percentage error.

#### Model Fitting and Diagnosis

**Figure 4:**
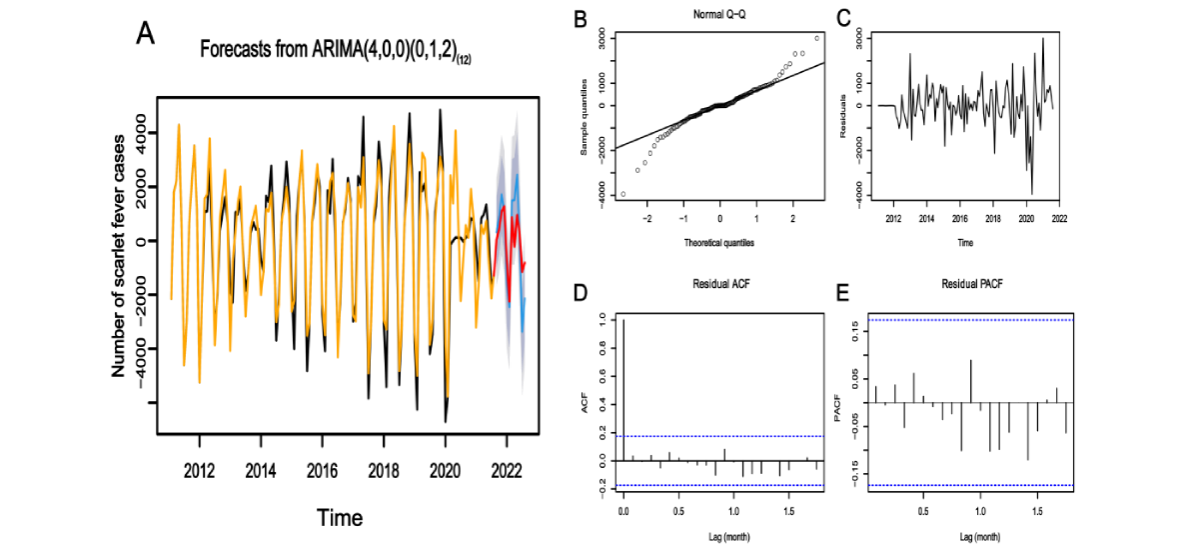
ARIMA(4,0,0)(0,1,2)_(12)_ model fitting, diagnosis, and prediction results. (A) Fitting and prediction results. Yellow and black are the fitted and true values of the training sets respectively. Red indicates the true values of the testing sets, and blue indicates the predicted values. (B) Normal Q-Q plot. The solid black line represents the theoretical quantile line. (C) Residual plot. (D) ACF plot. (E) PACF plot. The blue dashed lines in (D) and (E) indicate the confidence interval. ACF: autocorrelation function; ARIMA: autoregressive integrated moving average; PACF: partial autocorrelation function.

**Figure 5:**
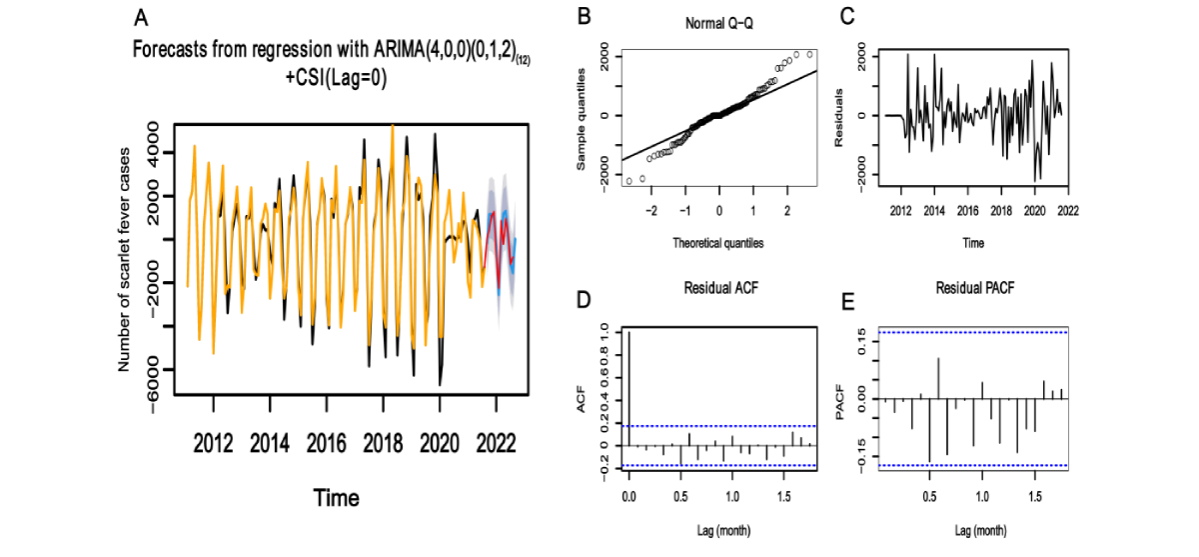
ARIMA(4,0,0)(0,1,2)_(12)_+CSI (Lag=0) model fitting, diagnosis, and prediction results. (A) Fitting and prediction results. Yellow and black are the fitted and true values of the training sets respectively. Red indicates the true values of the testing sets, and blue indicates the predicted values. (B) Normal Q-Q plot. The solid black line represents the theoretical quantile line. (C) Residual plot. (D) ACF plot. (E) PACF plot. The blue dashed lines in (D) and (E) indicate the confidence interval. ACF: autocorrelation function; ARIMA: autoregressive integrated moving average; CSI: comprehensive search index; PACF: partial autocorrelation function.

**Figure 6:**
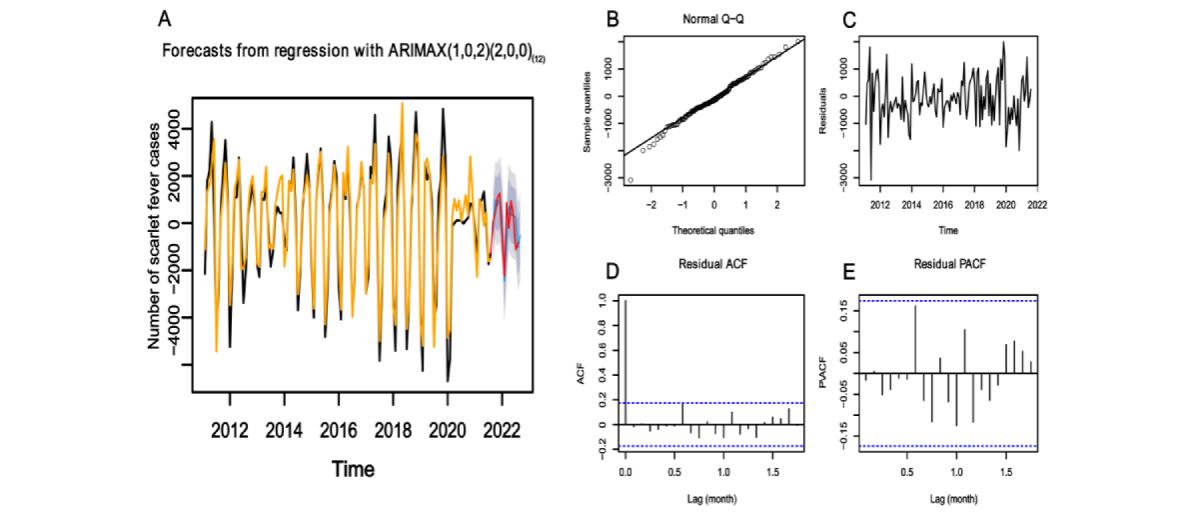
ARIMAX(1,0,2)(2,0,0)_(12)_ model fitting, diagnosis, and prediction results. (A) Fitting and prediction results. Yellow and black are the fitted and true values of the training sets respectively. Red indicates the true values of the testing sets, and blue indicates the predicted values. (B) Normal Q-Q plot. The solid black line represents the theoretical quantile line. (C) Residual plot. (D) ACF plot. (E) PACF plot. The blue dashed lines in (D) and (E) indicate the confidence interval. ACF: autocorrelation function; ARIMAX: autoregressive integrated moving average with explanatory variable; PACF: partial autocorrelation function.

Model 1 (Figure 4A), Model 2 (Figure 5A), and Model 3 (Figure 6A) showed good fitting performances with well-matched fitted and true values. Residual checking was conducted for Model 1 (Figure 4B-E), Model 2 (Figure 5B-E), and Model 3 (Figure 6B-E). The inspection of the residuals revealed the following results: Q-Q plots showed that the residuals followed a normal distribution, residual plots fluctuated around 0, and ACF and PACF plots were almost within the 95% CI. These findings confirmed that the models were suitable for the prediction session.

#### Model Prediction and Evaluation

The 3 models were used to forecast the incidence of scarlet fever with the testing sets. For Model 1 (Figure 4A), Model 2 (Figure 5A), and Model 3 (Figure 6A), the predicted and true values largely coincided, with the true values falling within the predicted interval. Comparing the performances of the models, the two ARIMAX models incorporating the BSI outperformed the ARIMA model, yielding lower MAE, RMSE, and MAPE values (Table 4).

**Table 4:** Evaluation of model prediction effectiveness.

Model	MAE^a^ (95% CI)	RMSE^b^ (95% CI)	MAPE^c^, % (95% CI)
ARIMA(4,0,0)(0,1,2)_(12)_	1692.16 (584.88-2799.44)	2036.92 (929.64-3144.20)	4.33 (0.54-8.13)
ARIMA(4,0,0)(0,1,2)_(12)_ + CSI (Lag=0)	1067.89 (402.02-1733.76)	1224.92 (559.04-1890.79)	3.36 (–0.24 to 6.96)
ARIMAX(1,0,2)(2,0,0)_(12)_	639.75 (188.12-1091.38)	830.80 (379.17-1282.43)	2.16 (–0.69 to 5.00)

^a^MAE: mean absolute error.

^b^RMSE: root mean square error.

^c^MAPE: mean absolute percentage error.

### Subgroup Analysis

In the original scarlet fever sequence, a significant decrease was noted in 2020. This decline may be attributed to the widespread outbreak of COVID-19, which could potentially have an impact on the model. To assess the impact of the pandemic on scarlet fever, models were constructed using training sets from 2011 to 2018. Subsequently, we predicted the incidence of scarlet fever in 2019, 2020, 2021, and from January to August 2022, then compared the predictions with the actual incidence. We employed a combination of the *auto.arima* function and ACF with PACF plots to construct the ARIMA(4,0,0)(2,1,0)_(12)_ and ARIMAX(0,0,3)(1,0,0)_(12)_ models based on the minimum AIC principle. The two models successfully passed the LSM (*P*<.001) and the residuals Ljung-Box (*P*=.93 and *P*=.87, respectively) tests. The fitting and predictive performances of the models are presented in Multimedia Appendix 5.

The results indicated that the predictive performances of both models declined in 2020 compared to the other years. However, the ARIMAX models, which incorporated the BSI, exhibited enhanced predictive capabilities and partially alleviated the impact of the pandemic.

## Discussion

Scarlet fever is highly prevalent among children aged 5-15 years. It was the primary cause of mortality among children in the 18th and 19th centuries [4]. This study revealed that, from 2011 to 2022, the monthly incidence of scarlet fever was 4462.17 (SD 3011.75) cases, with an annual incidence of 53,546.06 cases. The highest incidence was observed in 2019. Previous studies have demonstrated that scarlet fever underwent a global resurgence with a sharp rise in the United Kingdom, Germany, Korea, and China between 2011 and 2014 [28]. Compared to the preoutbreak level, the annual incidence of scarlet fever doubled in China [5], tripled in the United Kingdom [29], and quadrupled in Korea [30]. The resurgence of scarlet fever in China may be related to the rapid economic development, improved living standards, meteorological conditions, and genetic characteristics of the host population [31]. The relaxation of the two-child and three-child policies has increased in the susceptible population. The largest study on scarlet fever conducted by Liu et al [5] revealed that the elevated incidence of scarlet fever in northern China may be connected to the cold seasons, poor ventilation, and a gradual replacement of emm1 genotypes resulting in a reduction in cross-immunity. As scarlet fever is susceptible to previous cases, prioritizing the improvement of early warning and prediction systems is crucial. There is an urgent need to explore innovative monitoring methods.

The outbreak of infectious diseases has significant health implications and noticeably impacts health care systems and economies. Certain contagious diseases have high transmission rates and various modes of spread, making them prone to causing widespread epidemics. Thus, it is imperative to promptly identify and implement measures to curb their proliferation from the early stages. Conventional disease surveillance is inherently characterized by a degree of latency, whereas internet-derived data has the potential to offer real-time information, thus enabling the faster detection of early indicators of disease outbreaks. Eysenbach et al [32] pioneered a significant precedent in using internet-derived data for disease surveillance. Studies indicated that the BSI can be employed in the early warning and prediction of infectious diseases [15,16]. Therefore, we identified 6 keywords associated with scarlet fever. Among these keywords, those that were highly correlated with scarlet fever, such as “symptoms of scarlet fever” and “Is scarlet fever contagious?”, exhibited consistent temporal trends with scarlet fever. Their search volumes changed in accordance with variations in the prevalence of scarlet fever. Spearman rank correlation revealed a strong positive association between the aggregated CSI and scarlet fever (r_s_=0.881), indicating that the BSI can effectively capture variations in scarlet fever epidemics.

The ARIMA model features low computational complexity, is versatile across various data types, and offers high precision in short-term predicting. This study employed scarlet fever time series data to construct the ARIMA(4,0,0)(0,1,2)_(12)_ model, and incorporated the BSI as an external variable to construct the ARIMAX(1,0,2)(2,0,0)_(12)_ and ARIMA(4,0,0)(0,1,2)_(12)_ + CSI (Lag=0) models. The 3 models exhibited favorable fitting performances, and the ARIMAX models demonstrated superior predictive performances, whether established based on ACF and PACF plots or using the *auto.arima* function. The results were consistent with those for brucellosis [26] and dengue fever [11], demonstrating the effectiveness and predictive ability of the BSI in monitoring scarlet fever. The BSI provides real-time data that can be accessed promptly, monthly or even daily, whereas the data published by the Chinese official website often incurs delays of several months. These indicators may suggest an impending outbreak of scarlet fever when an unusual surge in vocabulary related to the disease is detected or when the predicted values computed by the model increase. Public health authorities can take proactive measures, prearrange prevention and control strategies, and allocate public health resources rationally.

The COVID-19 outbreak posed an immense threat to human life and resulted in a worldwide pandemic. The original time series of scarlet fever revealed a substantial decrease in incidence in 2020, potentially influencing the performance of the model. Due to the overlapping symptoms of COVID-19 and scarlet fever, we conducted subgroup analyses to assess the epidemic’s impact and address potential variations. The results exhibited a decline in the model’s predictive performances in 2020, consistent with the findings from Ma et al [2]. This decline could be attributed to the containment measures that were implemented, which may have disrupted the transmission pathways of the disease. Moreover, the ARIMAX model, which integrated the BSI, exhibited superior predictive performances, providing additional evidence for the successful use of the BSI concerning scarlet fever.

This study had several limitations. First, scarlet fever is influenced by a variety of factors, including meteorological conditions, sanitation, and population immunity. This study exclusively used the BSI for analysis, but other factors could be considered in future analyses. Second, internet-derived data is susceptible to the impact of media coverage, possibly resulting in the inclusion of inaccurate information [14]. Finally, Baidu search engines do not cover all internet users, and the keywords included in the BSI are not exhaustive. In the future, collaborative efforts with other search engines could be pursued to enhance the predictive capabilities of the model.

In conclusion, the BSI can be used as a valuable supplement to traditional surveillance systems, providing early warning and prediction capabilities for scarlet fever. Furthermore, it presents novel perspectives and provides a theoretical foundation for the early identification and prediction of infectious diseases, thereby facilitating the timely implementation of public health intervention measures.

## Appendix

Multimedia Appendix 1 Correlation between monthly Baidu search index keywords and reported cases of scarlet fever from January 2011 to August 2022.

Multimedia Appendix 2 Stationary series plots after first order differencing.

Multimedia Appendix 3 The CCF between scarlet fever and the comprehensive search index. Blue dashed lines indicate the 95% CI. CCF: cross-correlation function.

Multimedia Appendix 4 Parameter estimation of candidate models.

Multimedia Appendix 5 Evaluation of the model fit, diagnosis, and prediction. AIC: Akaike information criterion; LSM: least squares method; MAE: mean absolute error; RMSE: root mean square error; MAPE: mean absolute percentage error.

## Abbreviations

ACF: autocorrelation function
ADF: augmented Dickey-Fuller
AIC: Akaike information criterion
ARIMA: autoregressive integrated moving average
ARIMAX: autoregressive integrated moving average with explanatory variable
BSI: Baidu search index
CCF: cross-correlation function
CSI: comprehensive search index
LSM: least squares method
MAE: mean absolute error
MAPE: mean absolute percentage error
PACF: partial autocorrelation function
RMSE: root mean square error
