# Supplementary material for: Early Warning and Prediction of Scarlet Fever in China Using the Baidu Search Index and Autoregressive Integrated Moving Average With Explanatory Variable (ARIMAX) Model: Time Series Analysis
Source: J Med Internet Res. 2023 Oct 30;25:e49400. doi: 10.2196/49400 (PMC10644180; doi:10.2196/49400)
Supplement: Multimedia Appendix 1 [file jmir_v25i1e49400_app1.docx]

**Multimedia Appendix 1** Correlation between monthly Baidu search index keywords and reported cases of scarlet fever from January 2011 to August 2022

| **Categories** | **Keywords^a^** | **r_s_^b^** | ***P* Value** | **Search volume**  **mean (SD)** |
| --- | --- | --- | --- | --- |
| 猩红热综合类 (scarlet fever comprehensive category) | 猩红热是什么病 (What is scarlet fever?) | 0.424 | <.001 | 420.17 (385.13) |
|  | 猩红热 (scarlet fever) | 0.709 | <.001 | 1474.07 (610.15) |
| 猩红热病因类 (scarlet fever etiology category) | 链球菌感染 (streptococcal infections) | 0.375 | <.001 | 339.47 (101.82) |
| 猩红热症状类 (scarlet fever symptoms category) | 咽峡炎 (sore throat) | 0.131 | .124 | 601.06 (539.18) |
|  | 疱疹性咽峡炎 (herpetic sore throat) | 0.218 | .010 | 2750.14 (3353.51) |
|  | 扁桃体化脓 (tonsil suppuration) | 0.384 | <.001 | 924.79 (335.83) |
|  | 发热 (fever) | -0.006 | .943 | 809.37 (238.57) |
|  | 脱皮 (peeling) | 0.030 | .726 | 257.71 (30.39) |
|  | 咽喉肿痛 (swollen throat) | 0.423 | <.001 | 860.88 (370.64) |
|  | 咽痛 (pain in the throat) | -0.165 | .051 | 278.18 (45.23) |
|  | 皮疹 (rash) | 0.054 | .529 | 1262.07 (281.95) |
|  | 多性红斑 (erythema multiforme) | 0.218 | .010 | 370.03 (79.67) |
|  | 斑疹 (blemishes) | 0.06 | .482 | 313.39 (285.67) |
|  | 斑丘疹 (papular rash) | -0.142 | .093 | 407.67 (548.76) |
|  | 猩红热图片 (scarlet fever pictures) | 0.764 | <.001 | 223.60 (113.43) |
|  | 猩红热的症状 (scarlet fever symptoms) | 0.664 | <.001 | 90.13 (36.63) |
|  | 猩红热传染吗 (Is scarlet fever contagious?) | 0.840 | <.001 | 176.54 (88.31) |
|  | 猩红热症状 (symptoms of scarlet fever) | 0.815 | <.001 | 524.19 (343.12) |
|  | 传染性红斑 (infectious erythema) | 0.211 | .012 | 88.62 (20.51) |
|  | 猩红热症状图片 (scarlet fever symptoms pictures) | 0.715 | <.001 | 72.95 (50.18) |
|  | 草莓舌 (strawberry Tongue) | 0.008 | .926 | 447.03 (329.42) |
|  | 风湿热 (rheumatic fever) | -0.053 | .537 | 469.14 (99.10) |
|  | 湿疹 (eczema) | -0.061 | .477 | 9519.9 (3374.61) |
|  | 荨麻疹 (urticaria) | -0.163 | .054 | 9376.48 (3203.87) |
|  | 荨麻疹图片 (urticaria pictures) | 0.248 | .003 | 3168.47 (2342.12) |
|  | 荨麻疹症状 (urticaria symptoms) | 0.241 | .004 | 1334.54 (670.12) |
|  | 麻疹 (measles) | 0.213 | .011 | 1765.54 (814.32) |
|  | 麻疹的症状 (measles symptoms) | -0.184 | .029 | 394.09 (280.99) |
|  | 麻疹图片 (measles pictures) | 0.456 | <.001 | 610.47 (289.88) |
|  | 病毒性皮疹 (viral rash) | -0.019 | .828 | 449.43 (378.71) |
|  | 带状疱疹 (herpes zoster) | -0.126 | .139 | 7067.68 (3124.54) |
|  | 风疹 (rubella) | 0.390 | <.001 | 1647.24 (475.73) |
|  | 风疹图片 (rubella pictures) | 0.083 | .326 | 409.67 (235.36) |
|  | 婴儿急疹 (infant rash) | -0.055 | .517 | 576.78 (221.63) |
|  | 幼儿急疹 (toddler rash) | 0.008 | .929 | 3126.39 (1488.70) |
|  | 幼儿急疹图片 (toddler rash pictures) | 0.112 | .189 | 786.18 (306.46) |
|  | 奶藓 (milk moss) | 0.254 | .002 | 453.91 (155.71) |
|  | 系统性红斑狼疮图片 (pictures of systemic lupus erythematosus) | 0.086 | .314 | 144.66 (189.76) |
|  | 玫瑰疹 (rose rash) | -0.077 | .363 | 510.85 (193.68) |
|  | 紫癜 (purpura) | -0.018 | .830 | 2337.73 (1035.16) |
|  | 过敏性紫癜 (allergic purpura) | 0.480 | <.001 | 3441.13 (1484.90) |
|  | 神经性皮炎 (neurodermatitis) | 0.265 | .002 | 2503.85 (726.02) |
|  | 过敏性皮炎 (allergic dermatitis) | 0.240 | .004 | 1818.44 (552.07) |
|  | 川崎病 (kawasaki disease) | 0.031 | .720 | 2646.59 (1358.03) |
| 猩红热防治类 (scarlet fever prevention and treatment category) | 猩红热治疗 (scarlet fever treatment) | 0.521 | <.001 | 92.45 (60.47) |
|  | 猩红热预防 (scarlet fever prevention) | 0.595 | <.001 | 39.07 (41.48) |
|  | 猩红热吃什么药 (What medicines to take for scarlet fever?) | 0.632 | <.001 | 46.20 (44.40) |
|  | 青霉素 (penicillin) | 0.013 | .878 | 2096.33 (466.37) |
|  | 红霉素 (erythromycin) | 0.331 | <.001 | 1367.26 (227.43) |
|  | 阿奇霉素 (azithromycin) | 0.291 | <.001 | 4553.15 (2040.36) |
|  | 克林霉素 (clindamycin) | 0.182 | .032 | 1593.32 (415.25) |
|  | 多西环素说明书 (doxycycline instructions) | -0.033 | .701 | 183.03 (69.89) |

^a^ Categories and keywords were presented in a Chinese (English) format.

^b^r_s_: Spearman rank correlation.
