# Supplementary figures and images for: Early Warning and Prediction of Scarlet Fever in China Using the Baidu Search Index and Autoregressive Integrated Moving Average With Explanatory Variable (ARIMAX) Model: Time Series Analysis
Source: J Med Internet Res. 2023 Oct 30;25:e49400. doi: 10.2196/49400 (PMC10644180; doi:10.2196/49400)

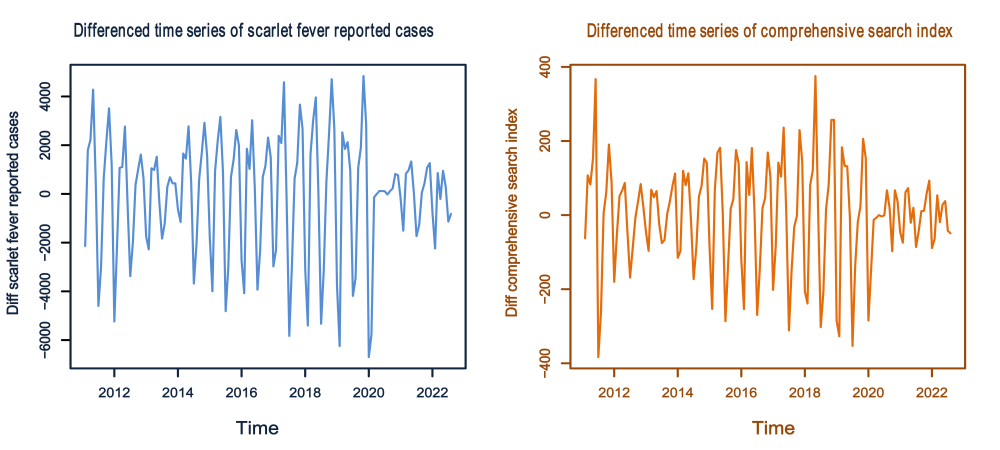

Supplement: Multimedia Appendix 2 [file jmir_v25i1e49400_app2.png]

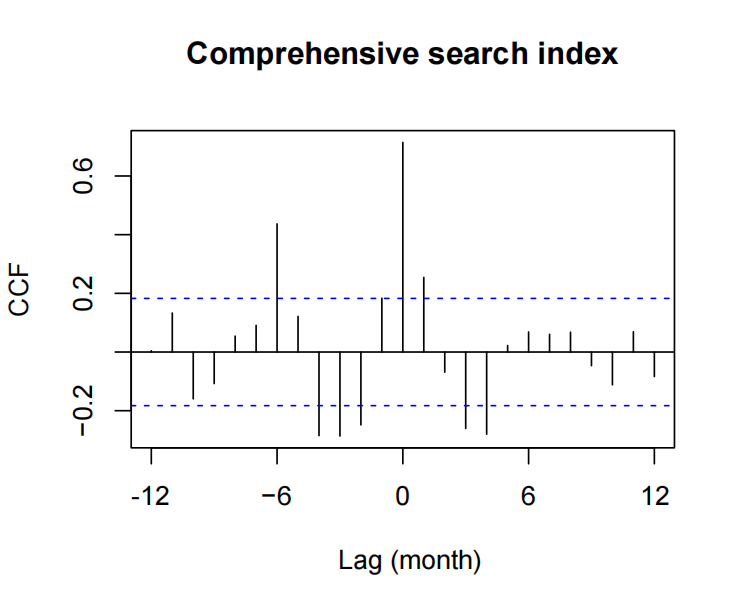

Supplement: Multimedia Appendix 3 [file jmir_v25i1e49400_app3.png]
