# Supplementary material for: Early Warning and Prediction of Scarlet Fever in China Using the Baidu Search Index and Autoregressive Integrated Moving Average With Explanatory Variable (ARIMAX) Model: Time Series Analysis
Source: J Med Internet Res. 2023 Oct 30;25:e49400. doi: 10.2196/49400 (PMC10644180; doi:10.2196/49400)
Supplement: Multimedia Appendix 4 [file jmir_v25i1e49400_app4.docx]

Multimedia Appendix 4 Parameter estimation of candidate models

| Model | Vars | Estimate | SE | Z value | Pr(>\|z\|) |
| --- | --- | --- | --- | --- | --- |
| ARIMA(4,0,0)(0,1,2)_(12)_ |  |  |  |  |  |
|  | AR1 | 0.343 | 0.089 | 3.870 | <.001 |
|  | AR2 | -0.468 | 0.097 | -4.803 | <.001 |
|  | AR3 | -0.004 | 0.094 | -0.041 | .967 |
|  | AR4 | -0.310 | 0.088 | -3.511 | <.001 |
|  | SMA1 | -0.689 | 0.212 | -3.244 | .001 |
|  | SMA2 | -0.311 | 0.125 | -2.479 | .013 |
| ARIMA(4,0,0)(0,1,2)_(12)_+CSI (Lag=0) |  |  |  |  |  |
|  | AR1 | 0.174 | 0.108 | 1.607 | .108 |
|  | AR2 | -0.334 | 0.099 | -3.369 | <.001 |
|  | AR3 | -0.022 | 0.094 | -0.231 | .817 |
|  | AR4 | -0.221 | 0.097 | -2.289 | .022 |
|  | SMA1 | -0.747 | 0.209 | -3.565 | <.001 |
|  | SMA2 | -0.147 | 0.156 | -0.945 | .345 |
|  | CSI(Lag=0) | 8.894 | 1.256 | 7.080 | <.001 |
| ARIMAX(1,0,2)(2,0,0)_(12)_ |  |  |  |  |  |
|  | AR1 | 0.557 | 0.112 | 4.994 | <.001 |
|  | MA1 | -0.589 | 0.123 | -4.790 | <.001 |
|  | MA2 | -0.387 | 0.116 | -3.333 | <.001 |
|  | SAR1 | 0.360 | 0.114 | 3.165 | .002 |
|  | SAR2 | 0.290 | 0.118 | 2.455 | .014 |
|  | CSI | 12.030 | 1.207 | 9.966 | <.001 |
| ARIMA(4,0,0)(0,1,1)_(12)_ |  |  |  |  |  |
|  | AR1 | 0.348 | 0.088 | 3.958 | <.001 |
|  | AR2 | -0.512 | 0.095 | -5.389 | <.001 |
|  | AR3 | -0.012 | 0.095 | -0.127 | .899 |
|  | AR4 | -0.323 | 0.088 | -3.662 | <.001 |
|  | SMA1 | -0.737 | 0.124 | -5.951 | <.001 |
| ARIMA(4,0,0)(0,1,1)_(12)_+CSI (Lag=0) |  |  |  |  |  |
|  | AR1 | 0.159 | 0.109 | 1.464 | .143 |
|  | AR2 | -0.322 | 0.100 | -3.256 | .001 |
|  | AR3 | -0.038 | 0.093 | -0.413 | .680 |
|  | AR4 | -0.212 | 0.096 | -2.200 | .028 |
|  | SMA1 | -0.762 | 0.123 | -6.168 | <.001 |
|  | CSI(Lag=0) | 9.235 | 1.222 | 7.556 | <.001 |
