# Supplementary material for: Early Warning and Prediction of Scarlet Fever in China Using the Baidu Search Index and Autoregressive Integrated Moving Average With Explanatory Variable (ARIMAX) Model: Time Series Analysis
Source: J Med Internet Res. 2023 Oct 30;25:e49400. doi: 10.2196/49400 (PMC10644180; doi:10.2196/49400)
Supplement: Multimedia Appendix 5 [file jmir_v25i1e49400_app5.docx]

Multimedia Appendix 5 Evaluation of the model fit, diagnosis and prediction

|  | Fit | | Diagnosis | | | Prediction | | |
| --- | --- | --- | --- | --- | --- | --- | --- | --- |
|  | AIC^a^ | R^2^ | LSM^b^  *P* value | Ljung-Box test | | MAE^c^ | RMSE^d^ | MAPE^e^, % |
|  |  |  |  | Chi-square | *P* value |  |  |  |
| ARIMA(4,0,0)(2,1,0)_(12)_  2019.1-2022.8 | 1357.64 | 0.92 | <.001 | 0.01 | .93 | 1783.66 | 2231.92 | 32.55 |
| 2019.1-2019.12 |  |  |  |  |  | 681.34 | 854.75 | 0.32 |
| 2020.1-2020.12 |  |  |  |  |  | 2345.39 | 2781.80 | 113.05 |
| 2021.1-2021.12 |  |  |  |  |  | 1958.91 | 2269.50 | 3.58 |
| 2022.1-2022.8 |  |  |  |  |  | 2331.70 | 2639.84 | 3.59 |
| ARIMAX(0,0,3)(1,0,0)_(12)_  2019.1-2022.8 | 1543.66 | 0.94 | <.001 | 0.03 | .87 | 652.23 | 913.80 | 12.40 |
| 2019.1-2019.12 |  |  |  |  |  | 821.03 | 908.48 | 0.37 |
| 2020.1-2020.12 |  |  |  |  |  | 914.09 | 1336.80 | 43.41 |
| 2021.1-2021.12 |  |  |  |  |  | 435.22 | 560.03 | 1.27 |
| 2022.1-2022.8 |  |  |  |  |  | 331.73 | 451.30 | 0.60 |

^a^AIC: Akaike information criterion.

^b^LSM: least squares method.

^c^MAE: mean absolute error.

^d^RMSE: root mean square error.

^e^MAPE: mean absolute percentage error.
